# Comparison of Accuracy of Whole-Exome Sequencing with Formalin-Fixed Paraffin-Embedded and Fresh Frozen Tissue Samples

**DOI:** 10.1371/journal.pone.0144162

**Published:** 2015-12-07

**Authors:** Ensel Oh, Yoon-La Choi, Mi Jeong Kwon, Ryong Nam Kim, Yu Jin Kim, Ji-Young Song, Kyung Soo Jung, Young Kee Shin

**Affiliations:** 1 Laboratory of Cancer Genomics and Molecular Pathology, Samsung Biomedical Research Institute, Samsung Medical Center, Seoul, Korea; 2 Institute for Refractory Cancer Research, Samsung Medical Center, Seoul, Korea; 3 Department of Pharmacy, College of Pharmacy, Seoul National University, Seoul, Korea; 4 Department of Pathology, Samsung Medical Center, Sungkyunkwan University College of Medicine, Seoul, Korea; 5 Samsung Advanced Institute for Health Sciences & Technology, Sungkyunkwan University School of Medicine, Seoul, Korea; 6 College of Pharmacy, Kyungpook National University, Daegu, Korea; 7 Research Institute of Pharmaceutical Sciences, College of Pharmacy, Kyungpook National University, Daegu, Korea; University of Navarra, SPAIN

## Abstract

Formalin fixing with paraffin embedding (FFPE) has been a standard sample preparation method for decades, and archival FFPE samples are still very useful resources. Nonetheless, the use of FFPE samples in cancer genome analysis using next-generation sequencing, which is a powerful technique for the identification of genomic alterations at the nucleotide level, has been challenging due to poor DNA quality and artificial sequence alterations. In this study, we performed whole-exome sequencing of matched frozen samples and FFPE samples of tissues from 4 cancer patients and compared the next-generation sequencing data obtained from these samples. The major differences between data obtained from the 2 types of sample were the shorter insert size and artificial base alterations in the FFPE samples. A high proportion of short inserts in the FFPE samples resulted in overlapping paired reads, which could lead to overestimation of certain variants; >20% of the inserts in the FFPE samples were double sequenced. A large number of soft clipped reads was found in the sequencing data of the FFPE samples, and about 30% of total bases were soft clipped. The artificial base alterations, C>T and G>A, were observed in FFPE samples only, and the alteration rate ranged from 200 to 1,200 per 1M bases when sequencing errors were removed. Although high-confidence mutation calls in the FFPE samples were compatible to that in the frozen samples, caution should be exercised in terms of the artifacts, especially for low-confidence calls. Despite the clearly observed artifacts, archival FFPE samples can be a good resource for discovery or validation of biomarkers in cancer research based on whole-exome sequencing.

## Introduction

Formalin fixing with paraffin embedding (FFPE) has been the standard sample preparation method used by pathologists, because FFPE tissues are stable at room temperature, easily storable, and suitable for large collections of clinical samples associated with historical records of disease progression and outcomes; however, FFPE tissues undergo extensive degradation and base alterations because of formalin fixation [1, 2]. Conversely, fresh frozen tissues are expensive to store, and difficult to collect for large-scale studies, but this approach minimizes the damage to nucleotides. Although the preferred specimen type for most molecular tests is the fresh frozen tissue, the use of FFPE samples is becoming popular in clinical studies because of the difficulties associated with the procurement of fresh frozen tissues in routine clinical practice. In particular, large-sized retrospective cancer studies for the evaluation of candidate biomarkers have been mostly conducted with FFPE samples; indeed, the wide variety of tumor samples preserved by using the FFPE method is a valuable resource for cancer research [3–6].

Increasingly, next-generation sequencing (NGS) is becoming the gold standard in cancer genomic research, and this revolutionary technique provides a comprehensive view of genomic alterations in tumors on the whole-genome scale at the nucleotide level. Recent reports opened up the possibility of application of NGS to DNA extracted from FFPE samples. Schweiger et al. showed that FFPE tumors can be used for NGS-based copy number variant (CNV) analysis, and Wood et al. expanded the NGS technique to generate a copy number karyogram using as little as 5 ng of DNA from FFPE samples on a multiplex platform [7, 8]. Kerick et al. demonstrated that FFPE tissues can supplement fresh frozen tissues in the detection of single nucleotide variants (SNVs) with 500 ng of input DNA; Yost proposed quality filters to identify high-confidence somatic mutations against the background of FFPE-induced base alterations [9, 10]. Spencer and colleagues performed targeted NGS for 27 cancer-related genes using paired frozen and FFPE samples to prove that FFPE samples can be equivalent to frozen samples for clinical NGS testing [11]. On the other hand, whole-exome sequencing (WES) data obtained from human prostate tissues uncovered discordance between FFPE tissue samples and their matched frozen tissue samples in terms of detection of SNVs and insertions or deletions (indels) at lower coverage levels (20×), while this discrepancy was reduced at higher coverage levels (>80×) [9]. Recently, comparison of WES data from 11 paired FFPE-frozen samples from lung adenocarcinoma patients were performed, and a reciprocal overlap of 90% somatic mutations was reported when considering the positions with sufficient sequencing depth [12].

In this study, we conducted an in-depth comparison of the WES data generated from 4 matched sets of FFPE and frozen tissue samples obtained from 4 cancer patients. The paired case study allowed us to directly compare the accuracy and concordance of the sequencing data obtained from the samples prepared by the 2 methods. For each set of paired samples, we analyzed the sequencing data yield, sequencing data quality, read alignments, insert sizes, the background sequencing error rate, the rates of FFPE-induced base alterations, and somatic mutation calls. During this analysis, DNA damage in FFPE samples was evident, but the sequencing data from FFPE samples were comparable to that for frozen samples.

## Materials and Methods

### Preparation of tissue samples

We obtained 4 pairs of matched FFPE-frozen tissue samples from 4 cancer patients (S1 Fig). Three (pair 1–3) of them were prepared by dissecting a tumor mass into 2 parts and by preserving each part by using 1 of the 2 methods. In each pair, matched blood or normal frozen sample was used for somatic mutation calling and comparison between 2 types of samples. Pair 4 was obtained from a patient who experienced cancer recurrence 3 times (in 2007, 2009, and 2010), and the 3 tumor samples were preserved by FFPE (in 2007 and 2009) and by freezing (in 2010). Pair 4 was excluded in comparing somatic mutation calls between matched FFPE and frozen samples. The frozen samples were preserved by fresh freezing in liquid nitrogen, and they were then stored at -80°C. The FFPE samples were fixed in a 10% formalin solution for 24 h at room temperature. After the fixation, the tissue samples were dehydrated using a series of graded ethanol solutions and were then treated with paraffin wax. The permeated tissue samples were then embedded in paraffin wax blocks. Blood samples from each patient were used as a control for the various assays. The study protocol was approved by the Institutional Review Board of the Samsung Medical Center. We obtained written informed consent from the donors regarding the use of their tissue samples in research.

### DNA library preparation, exome sequencing, and exome mapping

The extracted DNA samples from blood, frozen or FFPE tissue were analyzed using agarose-gel electrophoresis to assess the data integrity and any degradation (S2 Fig). Using Covaris, 3 μg of genomic DNA was randomly fragmented, and DNA fragments of approximately 200–300 bp were selected. Exome capture was performed using NimbleGen exome 2.1M array (pair 1 and 4) and SureSelect All Human exon V5 (pair 2 and 3), which targets ~30,000 coding genes (36.5 Mb and 50Mb target regions in NimbleGen and SureSelect respectively) in the CRCh37/Hg19 genome assembly. The captured DNA was sequenced on Illumina HiSeq 2000 machines, generating 2 × 100-bp paired-end reads. Sequencing data obtained from the Illumina pipeline were aligned with the UCSC hg19 assembly using the BWA (http://bio-bwa.sourceforge.net/) alignment algorithm.

### Analysis of WES data

#### Comparison of exome sequence data among blood, frozen, and FFPE samples

The sequence quality, mapping quality, sequencing depth, and insert sizes in the exome sequencing data were evaluated using SAM tools (http://samtools.sourceforge.net/), the statistical software R (http://www.r-project.org), and custom-made Perl scripts.

#### Estimation of the background sequencing error rate

To accurately measure base alteration rates induced by formalin fixation, sequencing errors or background DNA damage should be excluded. We used matched blood and normal frozen samples to estimate the background DNA damage/sequencing errors of the Illumina platform. Because we assumed that the DNAs from blood or fresh frozen samples were almost intact without artifacts, altered bases at homozygous sites in the samples were likely to be sequencing errors. There is a trade-off between ‘identifying altered alleles’ and ‘filtering sequencing errors’. If error-filtering is performed under highly stringent conditions (limited to high-quality reads/bases), true altered alleles could be missed. On the other hand, the error-filtering would not work properly if low quality bases are allowed. For the identification of homozygous sites, low error-filtering condition was applied to reduced the risk of missing true altered alleles, therefore, to ensure that the sites bear only a single type of allele. On the contrary, stringent error-filter was applied to in calling altered bases to ensure that the bases are not sequencing errors. The sequencing error rates were estimated as follows:

1. We labeled sites as *homozygous* in FFPE and frozen samples when the sites included only a single allele with mapping and base quality ≥20 and sequencing depth ≥50.
2. We labeled bases *discrepant* at the homozygous sites in the matched blood or normal frozen sample when the discrepant bases had mapping quality ≥60 and base quality ≥30.
3. In the blood/normal frozen samples, we calculated the sequencing error rate by dividing the number of discrepant bases by the total number of bases at the homozygous sites.

#### Estimation of the FFPE-induced rate of base alterations

Overall, base alteration (transition) rates were estimated in the way opposite to the sequencing error calculation. FFPE-induced base alteration rates were obtained by subtracting the sequencing error rates from the overall base transition rates.

1. We labeled sites *homozygous* when, in blood samples, mapping/base quality was ≥20 and sequencing depth was ≥50.
2. We labeled bases *discrepant* when mapping quality was ≥60 and base quality was ≥30 at the homozygous sites in matched frozen or FFPE samples.
3. We calculated overall base transition rates by dividing the number of discrepant bases by the total number of bases at the homozygous sites in frozen or FFPE samples.
4. We subtracted the rates of sequencing errors/background DNA damage from the overall base transition rates.

#### Comparison of SNV calls between frozen and FFPE samples

Somatic mutations were called for frozen and FFPE pairs of samples using MuTect [13]. *LOD*_T_ threshold of 6.3 and HC filters were applied to. Except the pair 4 which were not true matched samples, the overlapping fraction of somatic mutation calls between the paired FFPE-frozen samples were investigated by increasing *LOD*_T._ score from 6.3 to 250.

## Results

### The yield of sequencing data in FFPE samples

To test whether equal amounts of DNA from FFPE and frozen samples generate similar sequencing data, we compared a variety of parameters, including the number of reads, mapping results, and sequencing depth between the 2 types of samples. In all the pairs, genomic DNA samples extracted from FFPE tissues were severely degraded, whereas DNA from frozen tissues showed an obvious large band with only slight degradation (S2 Fig). Generally, the total yield and mapping rates were lower in FFPE samples, but the deterioration was not significant, except for FFPE-2 sample in pair 4, which appeared to have severe DNA damage and fragmentation. The FFPE samples yielded about 100 million unique reads on average, while about 150 million reads were generated for the paired frozen samples (Table 1). With the exception of FFPE-2 sample in pair 4, the mapping rates of the other FFPE were close to that of the frozen samples. The proportion of uniquely and properly paired reads (i.e., mapped in the correct orientation and within the insert size) was >98% in the frozen samples, and was about 90% in the FFPE samples (Table 1). The proportion of unmapped or discordantly mapped reads in the frozen samples was <1%, whereas the proportion of them for the FFPE samples was about 10% (Table 1).

**Table 1:** Whole-exome sequencing data statistics. FFPE: formalin-fixed paraffin-embedded samples.

	type	Unique Reads	Properly mapped reads	Discordantly mapped reads	Unmapped Reads	covered target region	On-target average depth	100-bp flanking region depth	% of off-target bases
pair 1	FFPE	106,062,549 (100%)	99,518,968 (93.8%)	3,474,934 (3.3%)	2,792,874 (2.6%)	96%	110×	59×	32%
	frozen	157,123,411 (100%)	154,986,824 (98.6%)	581,700 (0.4%)	1,467,405 (0.9%)	97%	105×	60×	63%
pair2	FFPE	127,734,224 (100%)	111,576,762 (87.4%)	7,202,130 (5.6%)	8,946,783 (7.0%)	98%	71x	22×	40%
	frozen	184,279,822 (100%)	181,875,732 (98.7%)	1,891,524 (1.0%)	498,601 (0.3%)	99%	152x	68×	48%
pair3	FFPE	115,195,059 (100%)	104,707,362 (90.9%)	4,864,588 (4.2%)	5,618,292 (4.9%)	98%	62x	18×	42%
	Frozen	156,655,931 (100%)	154,185,112 (98.4%)	1,940,720 (1.2%)	518,664 (0.3%)	99%	125x	55×	49%
pair4	FFPE-1	98,896,552 (100%)	87,624,296 (88.6%)	4,786,078 (4.8%)	5,785,040 (5.8%)	97%	77×	41x	41%
	FFPE-2	33,520,501 (100%)	12,795,876 (38.2%)	3,404,488 (10.2%)	16,622,576 (49.6%)	91%	9x	5x	41%
	frozen	89,170,706 (100%)	88,800,572 (99.6%)	143,020 (0.2%)	193,361 (0.2%)	97%	97x	60x	41%

Mean target coverage was significantly lower in the FFPE samples than in the matched frozen samples (Table 1). The discrepancy between the high mapping rate and low target depth was mainly due to the considerable number of soft clipped reads in the FFPE samples; we described it further in next section. Interestingly, the frozen samples showed higher off-target rates (40~60%) compared to the FFPE samples (30~40%; Table 1). The shorter DNA lengths in the FFPE samples increased the on-target coverage depth because the captured templates were not likely to extend far beyond the capture probes and were more likely to be within the target regions. In this sense, the off-target ratios of the FFPE samples were lower than those of the frozen tissue in all the pairs.

### DNA degradation in FFPE samples

**Fig 1:**
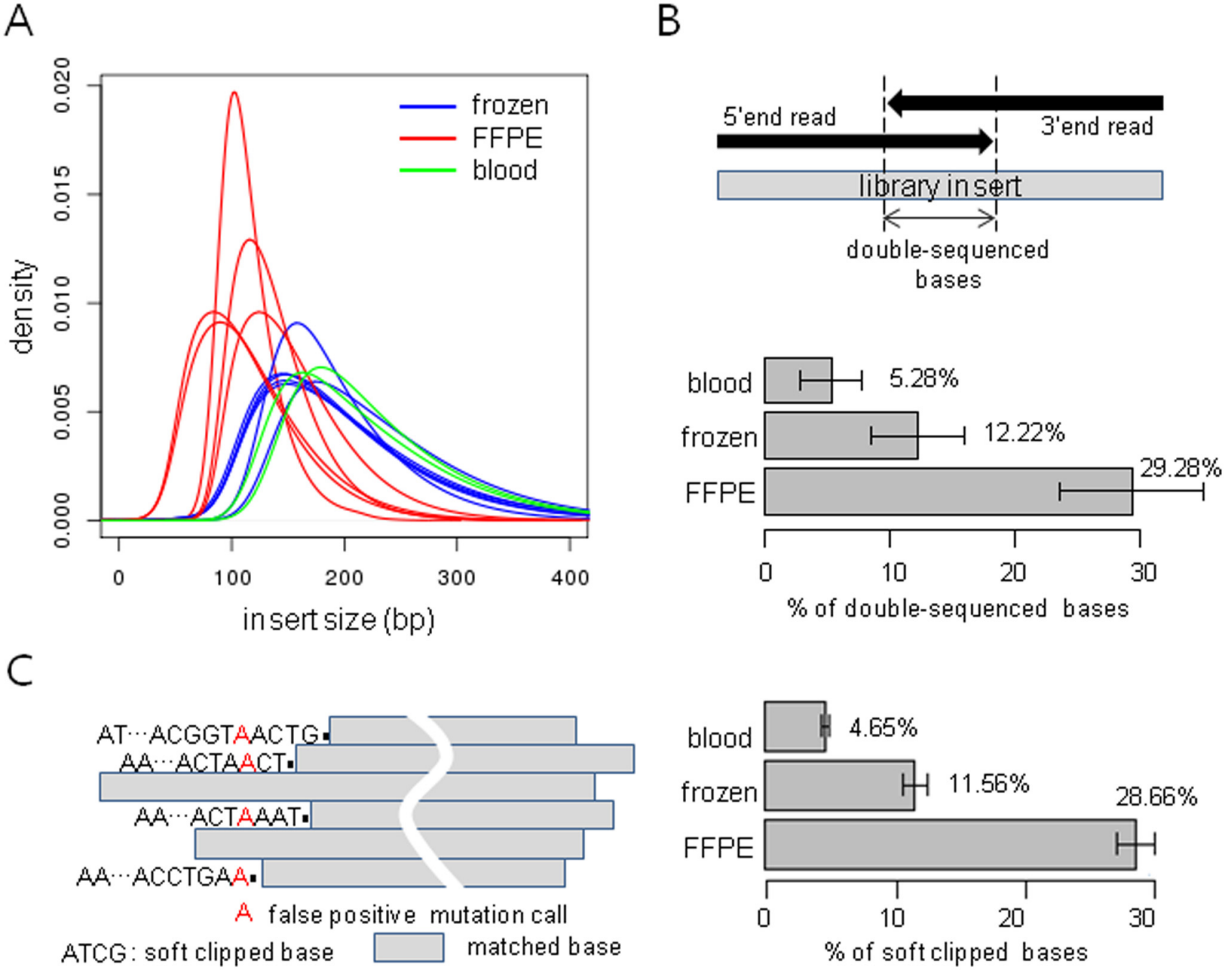
Distribution of insert sizes and frequencies of double-sequenced regions. The distribution of insert sizes was calculated from properly mapped paired reads. The distributions of formalin-fixed paraffin-embedded (FFPE) samples were skewed to the left because of a large number of short inserts (A). The short inserts generated abundant overlapping paired ends in FFPE samples (B), and soft clipped bases (C).

Formalin-induced DNA fragmentation is a well-known phenomenon, and we evaluated the extent of this effect by comparing library insert sizes determined using the distance between properly mapped paired reads. In all the pairs, FFPE samples showed shorter sequencing inserts than did the matched frozen samples. The median insert sizes of the frozen samples were about 150~200bp, while those of the matched FFPE samples were about 100 bp (Fig 1A). The amount of sequencing data in FFPE-2 sample in pair 4 was about 1/3 of the matched frozen, and the low yield of sequencing data could be due to the excessive fragmentation of the DNA templates, which were too short for bridge amplification on the Illumina platform [14].

Overlapping reads resulting from the short insert size could be problematic for variant calls on the paired-end sequencing platform (Fig 1B). If 2 paired-end reads overlapped, the bases in the overlapping positions were sequenced twice, doubling the frequency of these regions. For instance, if an insert was 100 bp and read length was 100 bp, then all of the bases in the insert will be sequenced twice by reading 100 bp from both ends of the insert. This situation may lead to false variant calls with overestimation of some alleles. The FFPE samples showed significantly greater numbers of double-sequenced bases compared to the frozen samples. Approximately 30% of the total sequencing data of the FFPE sample were double-sequenced by means of overlapping paired-end reads, and the proportion decreased to 10% and 5% in frozen and blood samples respectively (Fig 1B).

A large number of soft clipped reads in FFPE samples could be also problematic on the detection of somatic mutations. A soft clip is a special mismatch state in the alignment that is restricted to contiguous segments of the read at the 5’ or 3’ end (Fig 1C). About 30% of the reads in the FFPE samples were partially mapped with long overhangs (soft clipped bases), and it is probably due to the non-specific annealing between degraded DNA fragments during library construction (Fig 1C). Despite the sufficient sequencing yields, a considerable fraction of soft clipped reads lowered the target sequencing depth in the FFPE samples, and could lead to false positive mutation calls if the soft clipped bases are not masked. The effect of overlapping reads or soft clipped bases in FFPE samples would not be negligible when the sample has insufficient sequencing depth or low percentage of tumor cells. Therefore, caution should be exercised with the variant calls in FFPE samples, and the mutation callers implementing the filters that reduce the artifacts are highly recommended.

### Sequencing base quality in FFPE samples

**Fig 2:**
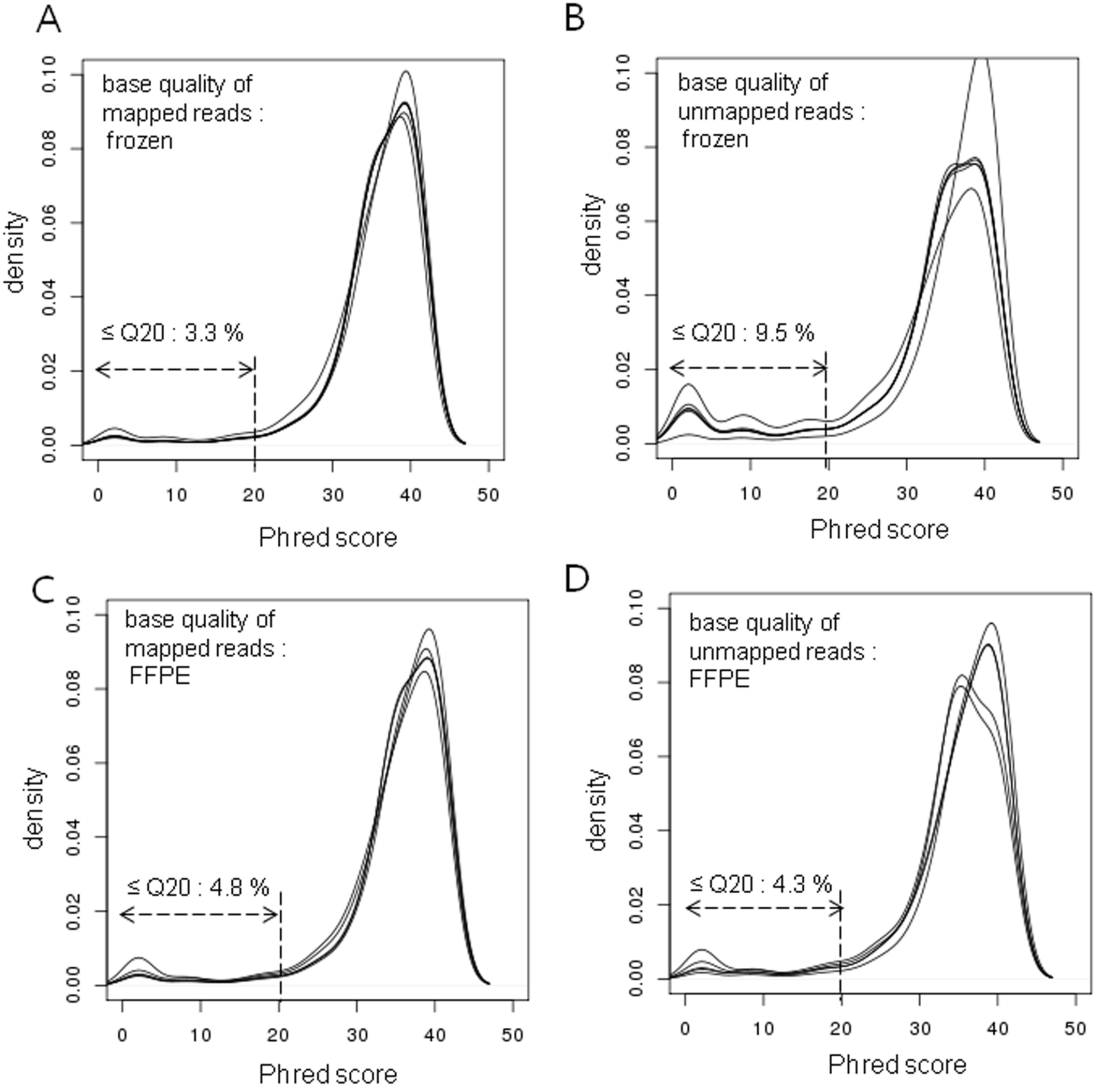
Distribution of nucleotide quality according to mapping status. The nucleotide quality scores were analyzed according to mapping status. In the mapped reads, there was no significant difference between the distributions of the 2 types of sample. In the unmapped reads, the blood and frozen samples showed a higher percentage of low-quality bases (≤ 20 on the Phred scale, black arrow) compared to the FFPE samples.

**Fig 3:**
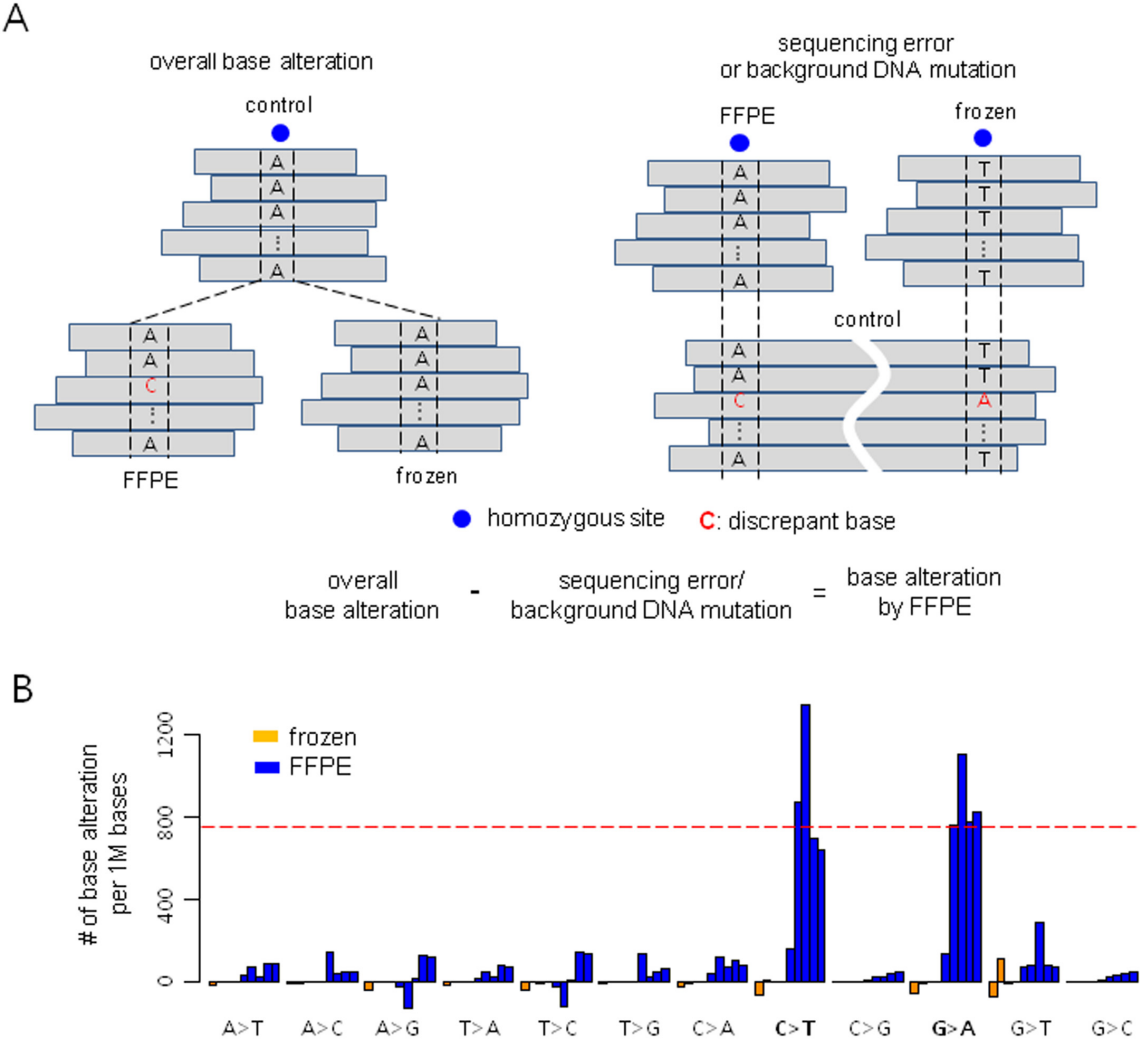
Frequency of base transition in formalin-fixed paraffin-embedded (FFPE) samples. **A**: A strategy for estimation of rates of sequencing errors/background DNA damage and overall base alteration rates. The discrepant bases at homozygous sites in control samples are likely to be sequencing errors or background DNA mutation. Conversely, discrepant bases at homozygous sites in frozen or FFPE tissue samples could be either sequencing errors/background DNA damage or base alteration caused by preservation methods. **B**: The rate of base alterations caused by formalin fixation. High frequencies of C>T and G>A were observed in FFPE tissue samples only.

We compared nucleotide quality of the sequencing reads generated from FFPE samples and from matched frozen samples. The sequencing quality of bases was analyzed according to their mapping status (mapped or unmapped). In the group of mapped reads, the nucleotide quality of the FFPE samples was as good as that in frozen samples; more than 80% of the bases in FFPE samples showed nucleotide quality ≥30 (on the Phred scale) in all the pairs, and there were no significant differences in the distributions of nucleotide quality between mapped and unmapped reads in FFPE samples (Fig 2A and 2B). However, the number of low-quality (≤20) bases doubled to around 10% in unmapped reads of frozen samples when compared to that of mapped reads (Fig 3C and 3D). In general, mapping failure occurs when alignments exceed the maximum number of mismatches allowed by mapping conditions. In blood and frozen samples, poor sequencing quality—likely to result in erroneous base calls—is expected to increase the number of mismatches. In contrast, the mapping failure in FFPE samples seemed to be caused not only by poor quality of base calling but also by base alterations before sequencing. Due to formalin fixation, base alterations occur before of sequencing, and the altered bases would result in mismatches in subsequent alignments, even though they are sequenced correctly. The small fraction of poor-quality bases in the unmapped reads of FFPE samples can be explained by the relatively large number of altered bases induced by formalin fixation.

### Base alterations caused by formalin fixation

It is well known that when compared to frozen tissue samples, FFPE samples have a high frequency of base alterations, mostly arising from formalin cross-linking of cytosines. As a result, during PCR, DNA polymerase fails to recognize the cytosine, then incorporates an adenine in place of a guanine, creating an artificial C>T or G>A mutation [2]. Here, we estimated the frequency of the base alterations specifically occurring in FFPE tissues.

First, the background DNA damage/sequencing error rate was measured by means of control normal samples. Generally, blood and frozen samples are believed to have intact DNA and to be free of tumor cells; therefore, the discrepancies at homozygous sites in blood/frozen DNA samples are likely to be background DNA damage or sequencing errors. The homozygous sites were selected from matched frozen tumor or FFPE tumor samples, and we counted the discordant bases at the homozygous sites in the control samples (Fig 3A). The frequency of each 12 possible types of base alteration was very similar ranging from 100~300 per Mb across the samples regardless of sample type, suggesting that the discrepancies were background DNA damage or sequencing errors (S1 Table).

Second, we estimated the frequency of overall base alterations (background DNA damage or sequencing errors + base alterations caused by preservation methods) that occurred in frozen or FFPE samples using opposite directions of analysis (Fig 3A). Discrepant bases in frozen or FFPE samples were counted at the homozygous sites identified in matched control samples; then, background DNA damage and sequencing error frequencies were subtracted from the overall base alteration frequencies. A high frequency of C>T and G>A alterations was uncovered in the FFPE samples (Fig 3C). The FFPE-specific base alteration rate varied among the FFPE samples (S2 Table). Pair 1 showed mild alterations, but pair 2–4 showed relatively severe alterations up to 1000 per Mb, especially in FFPE-2 of pair 4 which showed severe DNA fragmentation with the shortest insert size. In this analysis, we re-confirmed that C>T and G>A base transitions occurred specifically in the FFPE samples as formalin fixation artifacts.

### Somatic SNV calling

**Fig 4:**
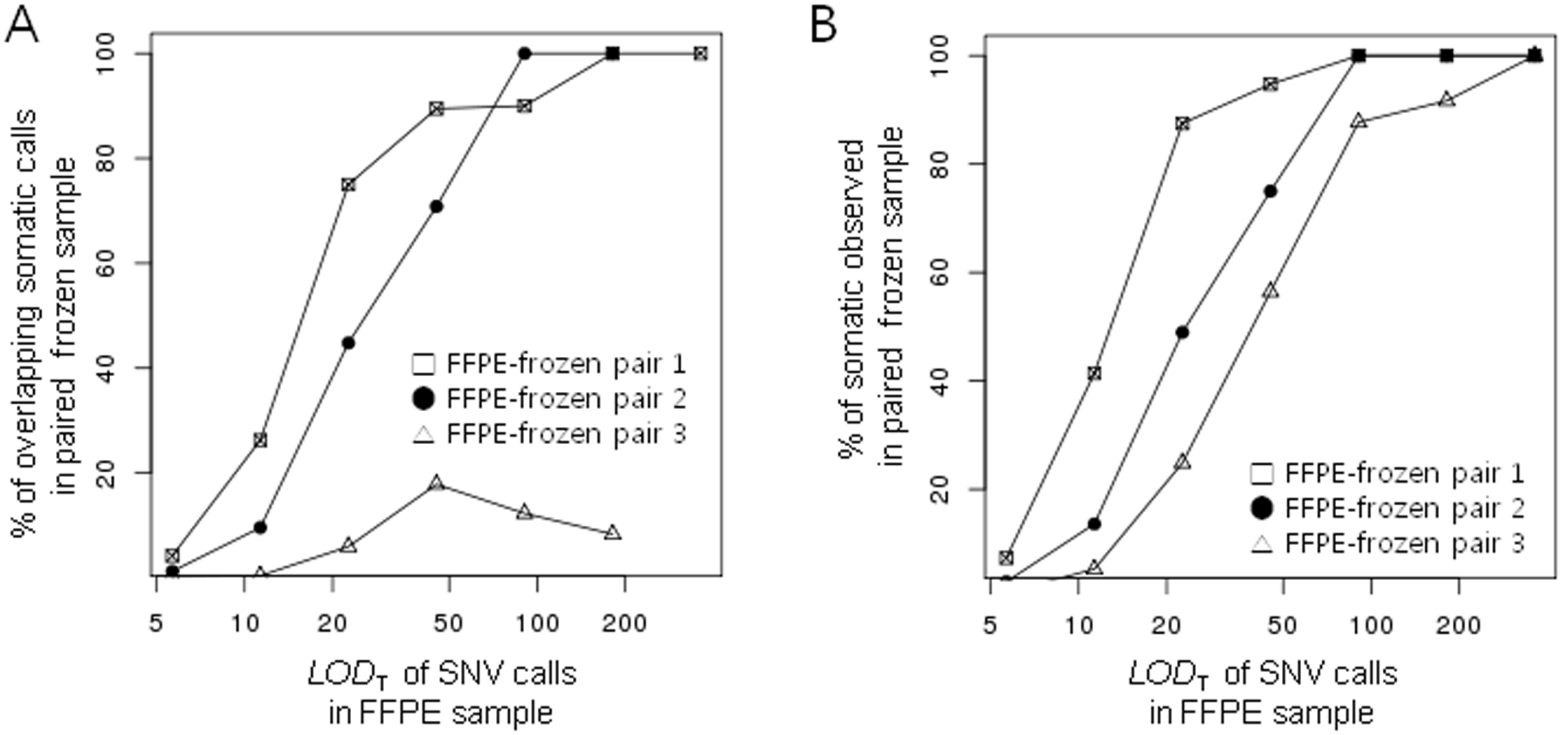
Comparison of somatic single nucleotide variant (SNV) calls. The overall concordance of somatic SNV calls between FFPE-frozen paired samples. A. The overlapping fraction of somatic mutation calls. B. The overlapping fraction of somatic mutation calls in FFPE samples when the matched frozen samples have at least one supporting read at the mutation.

We compared somatic mutation calls between frozen and matched FFPE samples because there is a possibility that the base alterations, double-counted or soft clipped reads could result in erroneous mutation calls. Pair 4 were excluded because the samples are not true FFPE-frozen pair which were obtained from recurrent tumors of a patient (in 2007, 2009, and 2010) and preserved by FFPE (in 2007 and 2009) or by freezing (in 2010). The discrepancies between the frozen and FFPE sample could involve different tumor cell subpopulations evolved in the course of repeated recurrence and chemotherapy [15]. We used MuTect which employed a Bayesian classifier to detect somatic mutations with very low allele fractions, and it implemented the filters for screening out various artifacts including overlapping reads and soft clipped bases. The likelihood of a somatic mutation event is interpreted by a *LOD*_T_ score (log odds in tumor), and the higher a *LOD*_T_ is, the more confident the mutation is. When we compared the somatic mutation calls with *LOD*_T_ cutoff of 6.3, total numbers of the calls in FFPE samples were at most 10 times as many as those called in the matched frozen samples mainly due to the high sensitivity of MuTect to the mutations with low allele frequencies. The discrepant mutation calls in FFPE samples could be either erroneous calls due to the artifacts arising from noisy sequencing data of the FFPE samples or true positive mutations which were not detected due to low allelic fraction in the matched frozen samples. There were few overlapping among the low confident calls (*LOD*_T_ < 10), however, the fraction of overlapping calls increased gradually as the *LOD*_T_ scores increased, and the concordance reached about 80% of the calls in matched frozen samples at *LOD*_T_ = 50 (Fig 4A). Pair 3 showed a poor concordance between the two types of sample regardless of *LOD*_T_ score because of the low percentage (about 10%) of tumor cells in the matched frozen sample. When we included the mutations that supported by at least one read in the matched frozen samples, the proportion of overlapping calls in pair 3 increased dramatically reaching about 60% at *LOD*_T_ = 50, whereas pair 1 and 2 showed a slight increase in the number of overlapping calls (Fig 4B). From the results, we speculated that low confident calls in FFPE samples are more likely to be false positive mutations which would not be observed in the matched frozen samples even with sufficient sequencing depth. Therefore, to reduce the effect of the artifacts in the sequencing data of FFPE samples, higher thresholds are required in FFPE samples. *LOD*_T_ > 50 would be a good cutoff for FFPE samples in case of MuTect.

## Discussion

NGS is a powerful tool for identifying genomic variants in cancer; in the clinic, the NGS technique combined with FFPE samples can be a good approach to cancer research or validation of biomarkers. In this study, we sequenced 4 sets of blood, frozen, and FFPE tissue samples using WES and conducted extensive comparison of the 2 types of samples to determine whether FFPE tissue samples are equally suitable for WES applications as frozen tissue samples. There were major differences between the 2 types of sample arising from formalin fixation and DNA fragmentation in FFPE samples. Firstly, the FFPE samples had significantly shorter inserts, which could result in double sequencing and lead to overestimation of certain alleles. Secondly, a considerable number of soft clipped reads was found in FFPE samples, and it could result in erroneous mutation calls, especially at the positions with insufficient sequencing depth. Thirdly, the base alterations C>T and G>A caused by formalin fixation were observed in FFPE samples, and the estimated alteration frequency increased up to 1/1000 bp in a heavily damaged sample.

After filtering out overlapping reads and soft-clipped bases, the FFPE samples exhibited quite reliable somatic SNV calls showing good concordance with matched frozen samples when considering confident calls. The nucleotide alterations caused by formalin fixation were expected to confound identification of somatic DNA variants; however, they had little on somatic mutation calls. The negligible effects on mutation calls can be explained by “random alteration” and “filtering by alignment.” Because the altered bases are distributed randomly across genomic DNA, heavily damaged regions are likely to be discarded from the alignment owing to excessive mismatches that cannot be allowed by the mapping algorithm. However, low sequencing depth can allow the alterations to become erroneous mutations calls, and Kerick et al. recommend high coverage of more than 80× to obtain high-confidence genomic information when working with FFPE samples [9]. Other authors also developed a filtering method for identification of high-confidence somatic mutations in the FFPE samples; their method involves removal of false positive calls caused by formalin fixation [10].

Currently, NGS is increasingly used in the clinic; generating WES data from archival FFPE tumor samples is a challenging task. FFPE samples frequently have historical records of disease progression and outcomes, and this information may become the focus of powerful retrospective studies. Nevertheless, most of the FFPE tissues are stored only for several years, whereas retrospective studies often include FFPE samples older than 10 years. There are several studies about the relationship between DNA quality and formalin fixation conditions such as ischemic time; however, is difficult to determine quality of DNA from FFPE samples before sequencing [7, 11]. In our study, all of the FFPE samples were prepared according to the same protocol, and all of them showed severe degradation when analyzed on agarose gels. We found that each sample had different levels of DNA damage—from mild to severe—when analyzing the sequencing data. Thus, objective criteria for measuring DNA quality in FFPE samples are needed for successful use of such tissue samples in WES.

In summary, our results demonstrated that DNA derived from routinely processed FFPE specimens produced NGS data that were similar in quality to DNA from frozen samples, and the data were informative too. FFPE samples are expected to become a good resource of genetic material for discovery or validation of biomarkers in NGS-based cancer research.

## Supporting Information

S1 Fig Schematic explanation of the tested samples. Four sets of matched frozen and formalin-fixed paraffin-embedded (FFPE) samples were obtained from cancer patients.
(TIF)

S2 Fig Agarose gel electrophoresis of genomic DNA extracted from frozen and formalin-fixed paraffin-embedded (FFPE) samples. Two hundred nanograms of genomic DNA from each sample was analyzed using electrophoresis in a 1% agarose gel. For comparison among FFPE samples, 1 μg of genomic DNAs was analyzed by electrophoresis.
(TIF)

S1 Table The rates of sequencing errors and background DNA damage. A sequencing error was defined as discrepant bases at a homozygous site in a control sample. Matched formalin-fixed paraffin-embedded (FFPE) or frozen samples were used to identify homozygous sites that consisted of a single allele with sufficient depth and base quality. Frequencies of all possible base transitions were estimated.
(PDF)

S2 Table Overall base transition rates. The base transitions (nucleotide alterations) that occurred in frozen or formalin-fixed paraffin-embedded (FFPE) samples were defined as discrepant bases at homozygous sites identified in a matched control sample. Overall, base transition rates include both sequencing errors/background DNA damage and preservation artifacts. C>T and G>A (red) occurred frequently in FFPE samples, and the other transitions (blue) showed similar frequencies across the samples regardless of the sample type.
(PDF)
