# Supplementary figures and images for: Comparison of Accuracy of Whole-Exome Sequencing with Formalin-Fixed Paraffin-Embedded and Fresh Frozen Tissue Samples
Source: PLoS One. 2015 Dec 7;10(12):e0144162. doi: 10.1371/journal.pone.0144162 (PMC4671711; doi:10.1371/journal.pone.0144162)

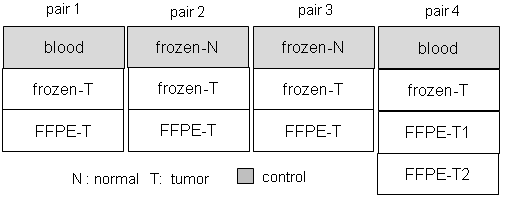

Supplement: S1 Fig — Four sets of matched frozen and formalin-fixed paraffin-embedded (FFPE) samples were obtained from cancer patients. (TIF) [file pone.0144162.s001.tif]

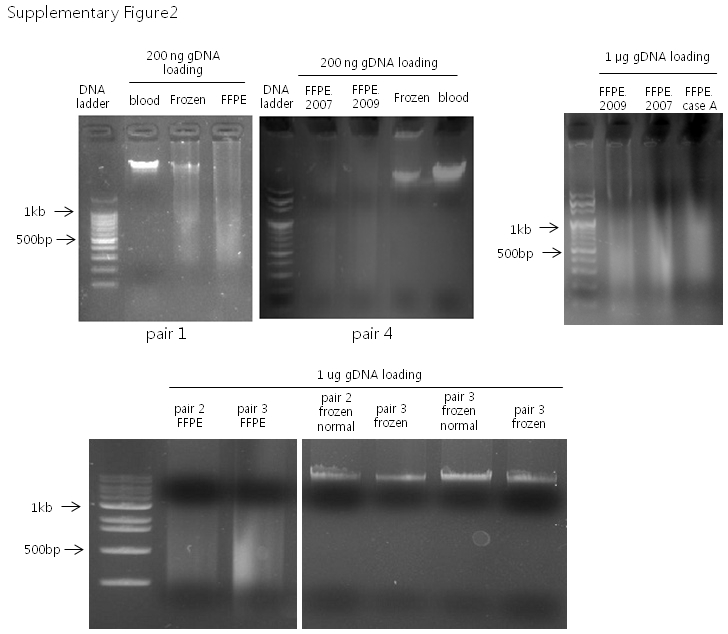

Supplement: S2 Fig — Two hundred nanograms of genomic DNA from each sample was analyzed using electrophoresis in a 1% agarose gel. For comparison among FFPE samples, 1 μg of genomic DNAs was analyzed by electrophoresis. (TIF) [file pone.0144162.s002.tif]
