# Supplementary material for: Comparison of Accuracy of Whole-Exome Sequencing with Formalin-Fixed Paraffin-Embedded and Fresh Frozen Tissue Samples
Source: PLoS One. 2015 Dec 7;10(12):e0144162. doi: 10.1371/journal.pone.0144162 (PMC4671711; doi:10.1371/journal.pone.0144162)
Supplement: S1 Table — A sequencing error was defined as discrepant bases at a homozygous site in a control sample. Matched formalin-fixed paraffin-embedded (FFPE) or frozen samples were used to identify homozygous sites that consisted of a single allele with sufficient depth and base quality. Frequencies of all possible base transitions were estimated. (PDF) [file pone.0144162.s003.pdf]

S1 Table The rates of sequencing error and background DNA mutation

	Pair 1		Pair 2		Pair 3		Pair 4		
type	FFPE	Frozen	FFPE	Frozen	FFPE	Frozen	FFPE-1	FFPE-2	Frozen
A > A	568,124,384 (99.97%)	610,219,420 (99.97%)	917,491,361 (99.98%)	1,550,813,655 (99.98%)	999,089,989 (99.98%)	1,854,367,376 (99.99%)	454,562,645 (99.97%)	18,232,370 (99.97%)	542,257,823 (99.98%)
**A > C**	15,163 (0.00%)	17,067 (0.00%)	18,567 (0.00%)	33,430 (0.00%)	13,466 (0.00%)	25,127 (0.00%)	14,904 (0.00%)	2,892 (0.02%)	78,453 (0.01%)
**A > G**	101,250 (0.02%)	111,375 (0.02%)	93,601 (0.01%)	152,350 (0.01%)	97,021 (0.01%)	165,692 (0.01%)	69,152 (0.02%)	615 (0.01%)	16,784 (0.00%)
**A > T**	44,743 (0.01%)	49,653 (0.01%)	45,184 (0.00%)	72,498 (0.00%)	48,026 (0.00%)	81,860 (0.00%)	29,727 (0.01%)	1,328 (0.01%)	34,775 (0.01%)
C > C	448,657,453 (99.96%)	510,894,004 (99.96%)	793,785,737 (99,98%)	1,465,922,276 (99.98%)	861,100,252 (99.98%)	1,630,173,530 (99.98%)	355,178,810 (99.97%)	22,016,631 (99.97%)	434,545,323 (99.97%)
**C > A**	66,388 (0.01%)	76,429 (0.01%)	72,573 (0.01%)	135,250 (0.01%)	81,600 (0.01%)	153,773 (0.01%)	49,007 (0.01%)	2,684 (0.01%)	58,993 (0.01%)
**C > G**	13,974 (0.00%)	16,372 (0.00%)	24,428 (0.00%)	44,832 (0.00%)	21,490 (0.00%)	37,933 (0.00%)	10,289 (0.00%)	625 (0.00%)	13,059 (0.00%)
**C > T**	110,372 (0.02%)	129,527 (0.03%)	87,469 (0.01%)	159,997 (0.01%)	91,733 (0.01%)	167,679 (0.01%)	63,605 (0.02%)	3,828 (0.02%)	76,877 (0.02%)
G > G	385,652,503 (99.88%)	450,124,400 (99.87%)	748,275,125 (99.98%)	1,416,533,731 (99.98%)	792,061,532 (99.98%)	1,570,268,411 (99.98%)	315,986,254 (99.87%)	21,213,950 (99.87%)	362,558,606 (99.86%)
**G > A**	89,738 (0.02%)	108,068 (0.02%)	95,350 (0.01%)	180,963 (0.01%)	98,492 (0.01%)	189,801 (0.01%)	50,993 (0.02%)	3,720 (0.02%)	58,225 (0.02%)
**G > C**	11,326 (0.00%)	13,608 (0.00%)	23,148 (0.00%)	43,452 (0.00%)	19,696 (0.00%)	36,353 (0.00%)	9,535 (0.00%)	604 (0.00%)	11,316 (0.00%)
**G > T**	379,853 (0.10%)	446,719 (0.10%)	50,625 (0.01%)	98,161 (0.01%)	54,323 (0.01%)	107,843 (0.01%)	365,884 (0.12%)	22,659 (0.11%)	429,345 (0.12%)
T > T	629,321,461 (99.97%)	677,113,274 (99.97%)	931,992,133 (99.98%)	1,577,261,851 (99.98%)	1,017,436,577 (99.98%)	1,884,606,886 (99.98%)	516,146,637 (99.98%)	33,597,556 (99.97%)	601,395,734 (99.98%)
**T > A**	48,018 (0.01%)	52,813 (0.01%)	47,037 (0.01%)	75,639 (0.00%)	50,850 (0.00%)	86,334 (0.00%)	32,782 (0.01%)	2,215 (0.01%)	37,713 (0.01%)
**T > C**	110,593 (0.02%)	122,829 (0.02%)	99,549 (0.01%)	162,498 (0.01%)	103,311 (0.01%)	176,021 (0.01%)	77,774 (0.02%)	1,225 (0.00%)	17,943 (0.00%)
**T > G**	19,232 (0.00%)	21,742 (0.00%)	19,570 (0.00%)	35,486 (0.00%)	14,244 (0.00%)	26,703 (0.00%)	16,091 (0.00%)	5,400 (0.02%)	86,584 (0.01%)
