# Supplementary material for: Comparison of Accuracy of Whole-Exome Sequencing with Formalin-Fixed Paraffin-Embedded and Fresh Frozen Tissue Samples
Source: PLoS One. 2015 Dec 7;10(12):e0144162. doi: 10.1371/journal.pone.0144162 (PMC4671711; doi:10.1371/journal.pone.0144162)
Supplement: S2 Table — The base transitions (nucleotide alterations) that occurred in frozen or formalin-fixed paraffin-embedded (FFPE) samples were defined as discrepant bases at homozygous sites identified in a matched control sample. Overall, base transition rates include both sequencing errors/background DNA damage and preservation artifacts. C>T and G>A (red) occurred frequently in FFPE samples, and the other transitions (blue) showed similar frequencies across the samples regardless of the sample type. (PDF) [file pone.0144162.s004.pdf]

S2 Table   Overall base alteration rates

	Pair 1		Pair 2		Pair 3		Pair 4		
type	FFPE	Frozen	FFPE	Frozen	FFPE	Frozen	FFPE-1	FFPE-2	Frozen
A > A	1,026,081,399 (99.97%)	802,597,534 (99.98%)	885,293,768 (99.95%)	2,280,742,194 (99.98%)	784,740,618 (99.96%)	1,996,763,263 (99.99%)	651,673,494 (99.97%)	83,428,312 (99.97%)	733,916,888 (99.98%)
**A > C**	27,295 (0.00%)	15,088 (0.00%)	60,306 (0.01%)	47,826 (0.00%)	51,378 (0.01%)	27,279 (0.00%)	112,489 (0.02%)	16,803 (0.02%)	103,046 (0.01%)
**A > G**	159,719 (0.02%)	115,542 (0.01%)	215,597 (0.02%)	215,224 (0.01%)	177,840 (0.02%)	175,831 (0.01%)	17,780 (0.00%)	3,821 (0.00%)	21,404 (0.00%)
**A > T**	110,033 (0.01%)	54,168 (0.01%)	129,669 (0.01%)	103,283 (0.00%)	118,406 (0.02%)	85,705 (0.00%)	90,025 (0.01%)	8,024 (0.01%)	45,174 (0.01%)
C > C	863,851,600 (99.94%)	666,185,787 (99.97%)	799,658,150 (99.89%)	2,060,305,853 (99.98%)	722,159,364 (99.90%)	1,744,479,863 (99.98%)	574,579,373 (99.86%)	78,853,894 (99.82%)	594,623,459 (99.97%)
**C > A**	163,403 (0.02%)	84,427 (0.01%)	159,366 (0.02%)	188,828 (0.01%)	133,155 (0.02%)	162,265 (0.01%)	146,612 (0.03%)	15,567 (0.02%)	75,237 (0.01%)
**C > G**	33,634 (0.00%)	19,226 (0.00%)	62,478 (0.01%)	61,707 (0.00%)	54,326 (0.01%)	39,447 (0.00%)	30,008 (0.01%)	4,147 (0.01%)	16,567 (0.00%)
**C > T**	348,436 (0.04%)	126,610 (0.02%)	665,392 (0.08%)	219,102 (0.01%)	559,833 (0.08%)	182,410 (0.01%)	606,191 (0.11%)	119,662 (0.15%)	102,755 (0.02%)
G > G	770,655,564 (99.85%)	612,465,498 (99.89%)	765,078,942 (99.88%)	1,981,863,857 (99.98%)	685,326,073 (99.88%	1,680,590,154 (99.98%)	518,758,218 (99.78%)	70,438,809 (99.73%)	521,164,371 (99.85%)
**G > A**	286,734 (0.04%)	112,228 (0.02%)	707,179 (0.09%)	24,6072 (0.01%)	666,778 (0.10%)	206,165 (0.01%)	478,331 (0.09%)	90,043 (0.13%)	81,051 (0.02%)
**G > C**	29,014 (0.00%)	16,334 (0.00%)	59,330 (0.01%)	59,424 (0.00%)	52,899 (0.01%)	38,697 (0.00%)	29,304 (0.01%)	4,259 (0.01%)	15,199 (0.00%)
**G > T**	817,543 (0.11%)	563,349 (0.09%)	117,486 (0.02%)	127,020 (0.01%)	99,870 (0.01%)	111,622 (0.01%)	641,694 (0.12%)	95,895 (0.14%)	674,713 (0.13%)
T > T	1,240,302,920 (99.97%)	939,146,055 (99.98%)	894,628,500 (99.95%)	2,323,444,128 (99.98%)	789,058,902 (99.95%)	2,029,958,162 (99.98%)	790,533,584 (99.97%)	103,579,608 (99.97%)	880,073,116 (99.98%)
**T > A**	110,979 (0.01%)	60,920 (0.01%)	124,593 (0.01%)	110,354 (0.00%)	105,888 (0.01%)	91,203 (0.00%)	85,974 (0.01%)	9,191 (0.01%)	52,713 (0.01%)
**T > C**	185,907 (0.01%)	132,817 (0.01%)	235,117 (0.03%)	228,569 (0.01%)	197,417 (0.03%)	186,402 (0.01%)	21,557 (0.00%)	4,508 (0.00%)	25,900 (0.00%)
**T > G**	37,300 (0.00%)	20,948 (0.00%)	63,400 (0.01%)	50,618 (0.00%)	53,802 (0.01%)	29,181 (0.00%)	134,440 (0.02%)	19,500 (0.02%)	124,857 (0.01%)
